# The Treatment of Insanity: Existing Defects and their Remedy

**Published:** 1907-06-01

**Authors:** 


### THE TREATMENT OF INSANITY.

### Existing Defects and their Remedy.

VII. HOW TO TRY TO FIND A CURE.

THIS is the question. Is it not worth while to attempt the cure of diseases that are not only dreadful to the individual, but also a heavy burden on the community? More than 1,300 cases of general paralysis occur in England and Wales every year. Supposing each case has a duration of three years, the discovery of a remedy for general paralysis would mean the abolition of two asylums of the largest size. The number of cases of acute insanity is not recorded in the official statistics, but it is certainly not less than the number of cases of general paralysis. If a remedy could be found for only half of them, it would relieve the rates to a similar extent. The suggestion that these diseases may be curable is not chimerical. It is not a proposal to square the circle or to discover perpetual motion. It is much more hopeful than the search for a cure for cancer; for of the origin and nature of cancer we are still almost wholly ignorant, but we know beyond dispute that insanity can be produced by poison; and that many cases of acute insanity and all cases of general paralysis are produced by poison is a certainty. Many cases can, and do, recover, and of the cases that do not recover a large proportion are of the same nature and character as those that do. They remain trembling for a certain time in the balance between recovery and death, or between recovery and permanence of the insanity, and it needs but a small matter to turn the balance in one direction or the other. Even of general paralysis some cases recover. If, therefore, it is justifiable to found an institute, and to conduct at great cost experiments for the cure of cancer, *à fortiori* is it justifiable to do the same for these cases of insanity.

This, then, is the position. Here is a disease more dreadful than cancer; a disease not only more dire in its effects on the individual, not only imposing a much heavier burden on the community, but one that menaces even more than cancer unborn generations. Yet, while cancer has occupied, and does occupy, the most strenuous labours of an army of workers, for insanity little is being done. This state of things is a reproach to medical science. It shouts and clamours for a remedy. For reasons that have been given it is not feasible to look to existing institutions for what is required. What is wanted, and what must be provided, is a hospital, in the proper sense of the term, for mental disorders. It should have wards for in-patients, and a department for out-patients. It should be fitted with the peculiarities of a lunatic asylum and with the appliances of a hospital. It should have its clinical and pathological laboratories, its resident staff and its visiting staff; its physicians and its surgeons. It should be a hospital in name and in deed for the active study and treatment of disease; not a residence for mentally affected persons until they get well or die. Its wards would be fed by its out-patient department, and its patients would be treated until they were restored to health, or until they were deemed irrecoverable and passed on to asylums. It would be a school, both undergraduate and post-graduate, in which physicians could be trained who would subsequently have charge of asylums, and from which a knowledge of mental disorders would be diffused throughout the medical profession. One great difficulty that besets the general hospital would be absent from such an institution; and another that besets the registered hospital would have to be carefully excluded. There would be no difficulty about competition with the general practitioner. The general practitioner does not treat cases of mental disorder, and is glad to get them off his hands. But if the patients were permitted to pay for their accommodation there would be the same temptation that there is in registered hospitals to receive first, and retain longest, those who paid the most. For this reason the hospital should be independent of any payments on behalf of patients.

This, then, is the scheme that we advocate, and for this scheme money ought to be, and must be found. It is not at all necessary that a large sum should be found at first. There are ways in which a thoroughly efficient beginning might be made on a scale small enough to be inexpensive, but large enough to be thoroughly trustworthy. So far as we know, there is nothing in the constitution of, for instance, St. Luke's Hospital which would prevent the addition of the necessary equipment and the necessary staff. Or a beginning could be made by setting aside a ward in one of the existing general hospitals for the trial of the experiment. Already there are out-patient departments for mental diseases at St. Thomas's and at Charing Cross Hospitals, which are doing excellent work. The devotion of a ward to the same purpose would need but a small outlay of money, and more wards could be incorporated in the scheme as its success became assured. At the present moment four of the wards in Charing Cross Hospital are closed for lack of funds. One of these could be remodelled for mental cases and maintained for a year for 20 patients at a cost of about £1,500. We have no authority for saying that such a scheme would be welcomed by the authorities of either of the institutions that have been mentioned. The concrete instances have been given only to show how easily practicable it is to form the commencement of such a plan as we have outlined. If it were found fertile it would form a very strong ground for an appeal to the charitable public for funds for a more ambitious scheme.

